# Genome‐Wide Analysis of Memory, Language, and Executive Function Reveals Domain‐Specific Genetic Architecture in Alzheimer Disease

**DOI:** 10.1002/alz70855_105459

**Published:** 2025-12-24

**Authors:** Kyle M Scott, Azra Emekci, Alzheimer's Disease Genetics Consortium (ADGC), Vanessa Aguiar‐Pulido, Farid Rajabli

**Affiliations:** ^1^ University of Miami Miller School of Medicine, Miami, FL, USA; ^2^ Pioneer High School, San Jose, CA, USA; ^3^ –, –, PA, USA; ^4^ Department of Computer Science, University of Miami, Miami, FL, USA, Miami, FL, USA; ^5^ John P. Hussman Institute for Human Genomics, University of Miami Miller School of Medicine, Miami, FL, USA

## Abstract

**Background:**

Alzheimer disease (AD) involves progressive decline in multiple cognitive domains, including memory, executive function, and language. The genetic underpinnings of these key endophenotypes remain incompletely understood. Variability in neuropsychological assessment batteries across studies has been a major barrier to reproducibility, underscoring the need for standardized measurements. We leverage the Phenotype Harmonization Consortium (PHC) dataset and National Alzheimer's Coordinating Center (NACC) genetic data to perform a genome‐wide association study (GWAS) of these three cognitive domains. Our goal is to clarify the contributions of specific genetic variants to distinct AD endophenotypes and thereby gain deeper insight into the neuropathological processes driving this disease.

**Method:**

We conducted a genome‐wide association analysis using data from 7,279 non‐Hispanic White participants from the NACC datasets. Cognitive performance was assessed using harmonized measures of executive function, language, and memory. We employed generalized linear mixed models controlling for age, sex, education, and three principal components to examine associations between genetic variants and cognitive performance

**Result:**

We evaluated known AD‐associated loci (Bellenguez et al. 2022) and 27 loci reached nominal significance (*p* < 0.05) in at least one cognitive domain. Of these, 8 were nominally significant in exactly one domain, 11 in two domains, and 7 (including *APOE4, APOE2, ABCA7, USP6NL, WNT3, CD2AP*, and *EPDR1*) in all three domains. Notably, *APOE4* displayed the strongest overall effects, with the stronger effect size on memory (BETA_mem = −0.28, *p* = 1.10×10^−58) and comparatively weaker yet still significant effects on language (BETA_lan = −0.14, *p* = 2.59×10^−19) and executive function (BETA_exf = −0.12, *p* = 2.30×10^−17). These results underscore both the broad influence of certain variants across multiple cognitive measures and variation in effect magnitude by domain.

**Conclusion:**

These findings enhance our understanding of the biological mechanisms that contribute to domain‐specific cognitive decline in AD, underscoring the distinct processes driving deficits in memory, language, and executive function. Such insights pave the way for more precise, syndrome‐focused interventions, ultimately guiding a precision medicine approach tailored to the heterogeneous clinical manifestations of AD.

